# Combined percutaneous–endoscopic puncture rendezvous technique for biliary–enteric anastomotic occlusion after pancreaticoduodenectomy

**DOI:** 10.1055/a-2299-1974

**Published:** 2024-04-24

**Authors:** Yi Wen, Lin Yang, Xiao Li, Xiao Ma, Yong Pang

**Affiliations:** 1 General Surgery, Peopleʼs Liberation Army General Hospital of Western Theater Command, Chengdu, China; 2 56711 College of Medicine, Southwest Jiaotong University, Chengdu, China

Biliary–enteric anastomosis strictures after pancreaticoduodenectomy are infrequent, and their complete occlusion is rare 1 . Herein, we present a patient with left bile duct occlusion at the biliary–enteric anastomosis after pancreaticoduodenectomy who was successfully treated using a combined percutaneous–endoscopic puncture rendezvous technique.

A 57-year-old man was admitted for recurrent abdominal pain. He had undergone pancreaticoduodenectomy for an intraductal papillary mucinous neoplasm 2 years previously. Magnetic resonance imaging revealed a dilated and obstructed left hepatic duct ( Fig. 1 ), for which a biliary stent was placed using a combined percutaneous–endoscopic puncture rendezvous technique, similar to that reported previously 2 .

**Fig. 1:**
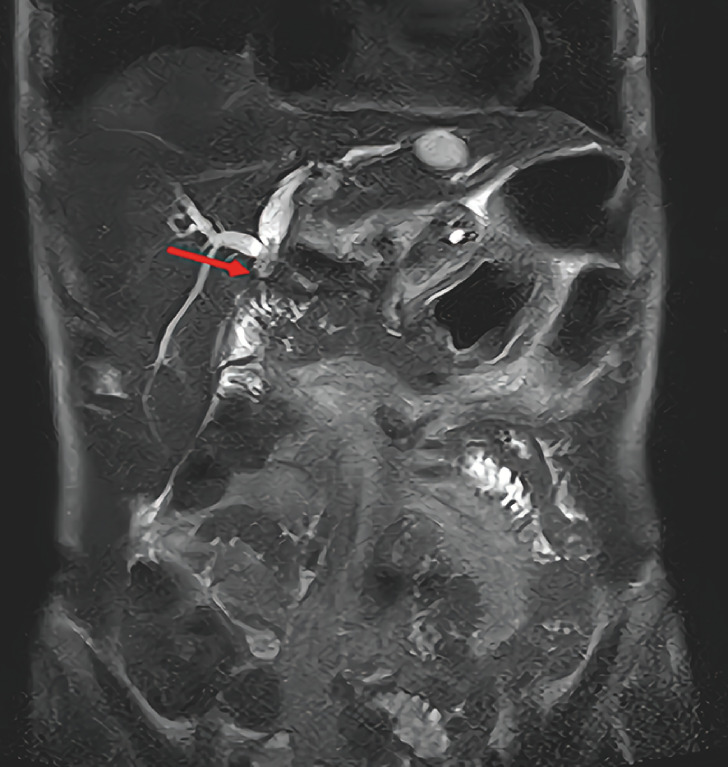
Magnetic resonance imaging showing the dilated and blocked left hepatic duct (red arrow) of a 57-year-old man with recurrent abdominal pain.

Ultrasound-guided percutaneous transhepatic biliary drainage was performed, followed by dilation of the drainage sinus up to 16 Fr 1 week later ( Fig. 2 , Fig. 3 ). Duodenoscopy showed a 2-mm anastomosis in the jejunal input loop, which did not communicate with the left hepatic duct. Choledochoscopy through the drainage sinus revealed that the opening of the left hepatic duct was closed due to scarring ( Video 1 ). Balloon occlusion cholangiography showed only the right bile duct ( Fig. 4 , Video 1 ).

**Fig. 2:**
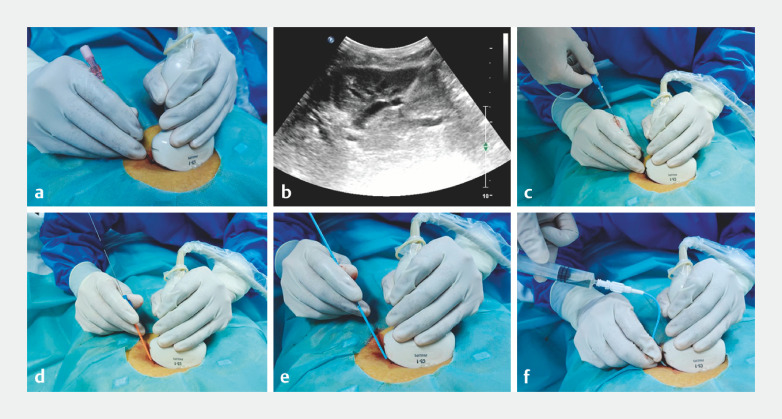
Ultrasound-guided percutaneous transhepatic puncture of the left extrahepatic bile duct. **a** The puncture site was determined under ultrasound guidance. **b** Ultrasound showing the puncture needle entering the bile duct. **c** Insertion of a metal wire. **d** Expansion of the puncture needle pathway. **e** Insertion of a 10-Fr pigtail drainage tube. **f** Extraction of bile.

**Fig. 3:**
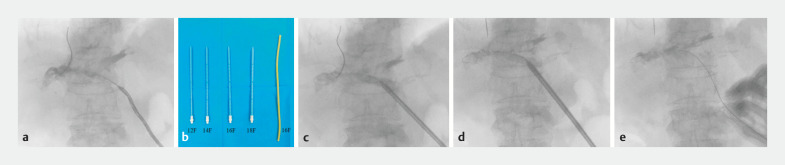
The drainage sinus was gradually dilated under fluoroscopic imaging. **a** Fluoroscopic imaging showing a zebra guidewire inserted through the pigtail drainage tube. **b** Expansion catheters (12F, 14F, 16F, and 18F) and a rubber drainage tube (16F). **c, d** Expansion catheters were inserted through the guidewire to dilate the drainage sinus under fluoroscopic monitoring. **e** Insertion of a 16-Fr rubber drainage tube.

**Fig. 4:**
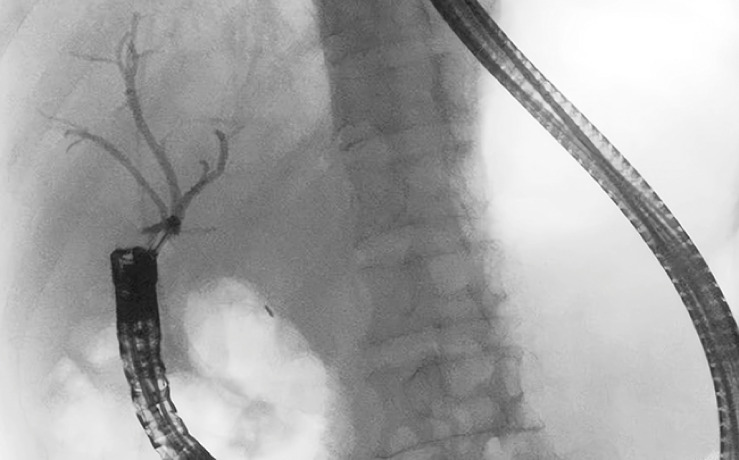
Balloon occlusion cholangiography showing only the right bile duct.

Treatment of a complete biliary anastomotic stricture that developed after pancreaticoduodenectomy using a combined percutaneous–endoscopic puncture rendezvous technique.Video 1

The mucosal injection needle was inserted into the scar through the working channel of the choledochoscope toward the transillumination from the duodenoscope. While withdrawing the mucosal injection needle, a bow cutting knife was positioned to insert a guidewire into the bile duct path created by the needle. Subsequently, the needle knife was used to cut the scarred area at the anastomosis and establish a new tract ( Video 1 ). Finally, a fully covered self-expandable metallic stent (60 × 10 mm) and a plastic biliary stent (8.5 Fr × 70 mm) were deployed across the anastomosis and extended to the distal left hepatic duct using the guidewire ( Fig. 5 ).

**Fig. 5:**
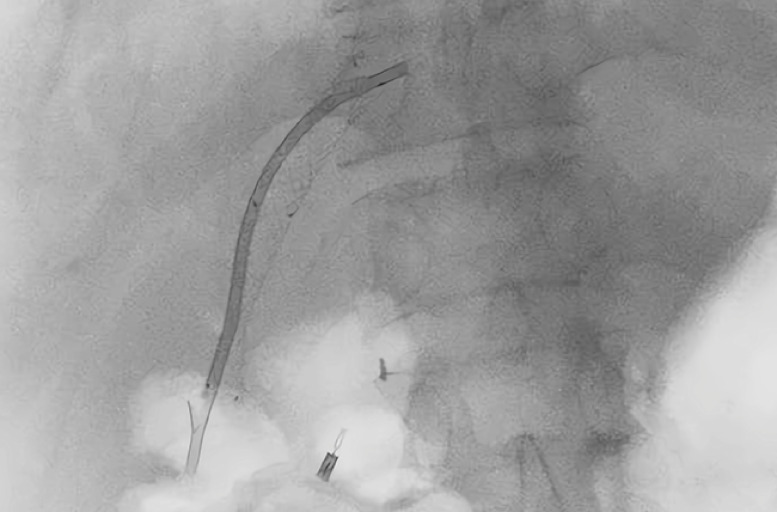
Fluoroscopic images showing the location of the biliary stents

For patients unsuitable for reoperation, the combined percutaneous–endoscopic puncture rendezvous technique is safe and effective for the treatment of refractory benign biliary–enteric anastomotic stenosis.

Endoscopy_UCTN_Code_TTT_1AR_2AG

## Notes

Endoscopy E-Videos https://eref.thieme.de/e-videos
*E-Videos* is an open access online section of the journal *Endoscopy* , reporting on interesting cases and new techniques in gastroenterological endoscopy. All papers include a high-quality video and are published with a Creative Commons CC-BY license. Endoscopy E-Videos qualify for HINARI discounts and waivers and eligibility is automatically checked during the submission process. We grant 100% waivers to articles whose corresponding authors are based in Group A countries and 50% waivers to those who are based in Group B countries as classified by Research4Life (see: https://www.research4life.org/access/eligibility/ ). This section has its own submission website at https://mc.manuscriptcentral.com/e-videos .
